# The emerging role of biosynthetic gene clusters in plant defense and plant interactions

**DOI:** 10.1371/journal.ppat.1009698

**Published:** 2021-07-02

**Authors:** Guy Polturak, Anne Osbourn

**Affiliations:** Department of Biochemistry and Metabolism, John Innes Centre, Norwich, United Kingdom; THE SAINSBURY LABORATORY, UNITED KINGDOM

## Introduction

The plant kingdom produces a diverse array of chemicals, collectively making an estimated 10^5^ to 10^6^ different metabolites [1,2]. These compounds are either known or likely to have important ecological functions, for example, in providing protection against herbivores, pests, and pathogens; in allelopathy (competition with neighboring plants); and in shaping the plant microbiome. In some cases, they have also been shown to function as regulators of plant growth and defense as well as primary metabolites sensu lato [3]. Plant natural products are formed by a series of enzyme-mediated chemical reactions that together constitute biosynthetic pathways. While it is well known that the genes for some well-characterized plant natural product pathways are dispersed throughout the genome, the last 2 decades have revealed a growing number of examples in which the genes for specific biosynthetic pathways are co-localized in plant genomes in biosynthetic gene clusters (BGCs). Several comprehensive reviews covering the nature and general features of plant BGCs have been published previously [4–8]. However, there has not as yet been a focused review of the roles of these clusters in the context of plant defense and plant interactions. Here, we review this topic, highlight major recent advances in the field, and discuss potential implications for crop improvement.

## Gene clustering occurs for diverse plant specialized metabolic pathways

The plant BGCs characterized to date range in size from tens to several hundred kilobases and typically contain 3 to 10 (for the most part) nonhomologous genes that participate in a shared biosynthetic pathway. An arbitrary definition of 3 genes as the minimal requirement for a plant BGC has been adopted for algorithm-based genome mining purposes, since the signal-to-noise ratio if 2 genes were used as the threshold level for predicting BGCs would be high [9]. Clearly, clustered pairs of nonhomologous but functionally related genes also exist in plant genomes and may together confer selective advantages. Examples include clustered pairs of terpene synthases and cytochrome P450s, e.g., for the biosynthesis of the phytoalexin capsidiol in pepper [10]. Such pairing of terpene synthases and cytochrome P450s is prevalent in multiple plant genomes [11]. Interestingly, pairing of protein functionality in plant defense can also occur in the form of fusion of functional domains within a single protein; nucleotide-binding leucine-rich repeat (NLR) proteins, involved in pathogen recognition, can be fused with various protein domains that serve as baits for pathogen effectors [12]. Some plant BGCs are highly compact, while others contain intervening genes and/or are more fragmented. The biosynthetic pathway genes encoded within BGCs are typically co-expressed, a feature that can be used as an additional criterion for identifying promising new clustered pathways [9,13,14].

**Fig 1:**
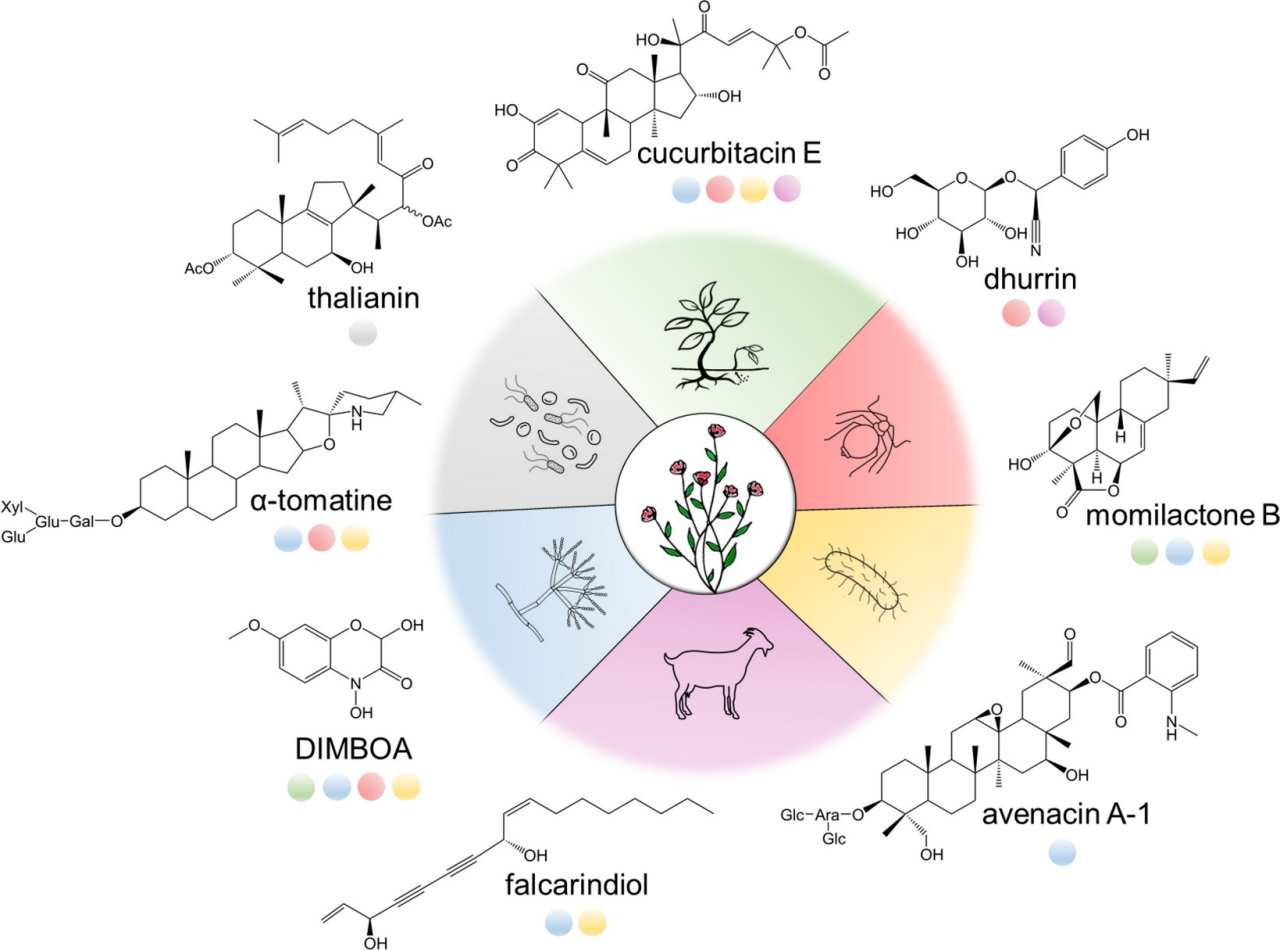
Examples of plant-specialized metabolites produced by BGCs and their roles in defense or other biotic interactions. Activities associated with each compound are depicted with color coding. From top, clockwise: allelopathy (green), insecticidal (red), antibacterial (yellow), anti-herbivore (purple), antifungal (blue), and modulation of microbiome (gray). BGC, biosynthetic gene cluster.

While BGCs are less prevalent in plants than in bacteria or fungi [15], it is now clear that the phenomenon of gene clustering in plant specialized metabolism is not rare or exceptional, with over 30 BGCs reported to date from distant phylogenetic clades across the plant kingdom, from both lower and higher plants. They encompass diverse classes of compounds, including terpenoids, alkaloids, fatty acids, polyketides, and cyanogenic glycosides, which exhibit activity against various types of pests and pathogens, including bacteria, fungi, insects, and herbivores, as well as against competing plants (Table 1 and Fig 1). These examples include defense compounds that are preformed (phytoanticipins) or produced in response to biotic stress (phytoalexins), as well as compounds that confer resistance to abiotic stresses (e.g., components of leaf waxes, which protect against desiccation). The specialized metabolites encoded by these BGCs have diverse modes of action, for example, disrupting pathogen cell membranes [16], conferring bitterness or toxicity that deters herbivores [17,18], undergoing pathogen-induced degradation to give bioactive volatiles [19], or forming physical barriers against biotic and abiotic stress factors [20]. Compounds produced by BGCs have also been shown to have other roles in interactions between plants and the environment, such as modulation of the root microbiome [21], although the consequences of this for plant growth and fitness are not yet known.

**Table 1:** Examples of characterized plant BGCs, and where known, their involvement in defense, and other ecological-related roles.

Compound(s)/pathway	Class	Plant species	Role in plant	Reference for BGC	No. of functionally characterized genes in BGC
Avenacins	triterpenes	*Avena* sp.	antifungal	[69] [48]	12
Arabidiol/arabidin	triterpenes	*Arabidopsis thaliana*	anti-oomycete, microbiome modulation	[70] [21]	3
Thalianol/thalianin	triterpenes	*Arabidopsis thaliana* *Arabidopsis lyrata*	microbiome modulation	[71] [21] [39]	5
Marneral	triterpenes	*Arabidopsis thaliana*	unknown	[70]	2
Tirucallol	triterpenes	*Capsella rubella*	unknown	[39]	5
Euphol	triterpenes	*Brassica rapa*	unknown	[39]	3
Cucurbitacins	triterpenes	*Cucumis sativus* *Cucumis melo* *Citrullus lanatus*	antibacterial, antifungal, insecticidal, anti-herbivore	[18] [30]	3
Yossosides	triterpenes	*Spinacia oleracea*	unknown	[50]	2
20-Hydroxy-betulinic acid	triterpenes	*Lotus japonicus*	unknown	[53]	2
Momilactones	diterpenes	*Oryza* sp. *Echinochloa crus-galli* *Calohypnum plumiforme*	antibacterial, antifungal, allelopathic	[24] [26]	4
Phytocassanes/oryzalides	diterpenes	*Oryza sativa*	antibacterial, antifungal	[72]	5
Casbene diterpenoids	diterpenes	*Ricinus communis* *Euphorbia peplus* *Jatropha curcas*	antifungal, antibacterial	[27]	7
5,10-Diketo-casbene	diterpenes	*Oryza sativa*	antifungal, antibacterial	[28]	3
Various monoterpenes and diterpenes	diterpenes/ monoterpenes	*Solanum* sp.	antibacterial, antifungal	[36]	3
Lycosantanolol	diterpenes	*Solanum lycopersicum*	unknown	[73]	3
α-Tomatine	steroidal glycoalkaloids	*Solanum lycopersicum*	antibacterial, antifungal, insecticidal	[31]	6
α-Solanine α-Chaconine	steroidal glycoalkaloids	*Solanum tuberosum*	antibacterial, antifungal, insecticidal	[31]	4
Noscapine	benzylisoquinoline alkaloids	*Papaver somniferum*	unknown	[47]	10
Thebaine	benzylisoquinoline alkaloids	*Papaver somniferum*	unknown	[74]	5
Hydroxycinnamoyl-tyramine conjugates	phenolamides	*Oryza sativa*	antibacterial, antifungal	[23]	4
Dhurrin	cyanogenic glucosides	*Sorghum bicolor*	insecticidal, anti-herbivore	[29]	3
Linamarin Lotaustralin	cyanogenic glucosides	*Lotus japonicus* *Manihot esculenta*	insecticidal, anti-herbivore	[29]	4
α-/β-/γ-Hydroxynitrile glucosides	hydroxynitrile glucosides	*Hordeum vulgare*	unknown	[75]	6
Falcarindiol	fatty acids	*Solanum lycopersicum*	antifungal, antibacterial	[60]	4
β-Diketones	polyketides	*Hordeum vulgare* *Triticum turgidum*	forming physical barrier on leaf surface	[20]	3
DIBOA/DIMBOA	benzoxazinoids	*Zea mays*	antibacterial, antifungal, insecticidal, allelopathic	[67]	7
Various acylsugars	acylsugars	*Solanum* sp.	antifungal, insecticidal, anti-herbivore	[66]	2

BGC, biosynthetic gene cluster.

BGCs have not been identified for some prominent groups of plant natural products (e.g., carotenoids and glucosinolates). For phenylpropanoids, a large, structurally diverse, and widely distributed class of compounds that includes many defense-related molecules [22], a first BGC has only recently been reported [23]. However, multispecies in silico analysis has predicted the existence of phenylpropanoid clusters in plant genomes in similar numbers to those of terpenoids and alkaloids [14]. It is not yet known why the biosynthetic genes for some types of compound are clustered in plant genomes and others are not. This may become clearer as the number of available plant genome sequences and characterized plant natural product pathways increases, and we learn more about the distribution, nature, and raison d’etre for plant BGCs.

In some cases, BGCs for closely related compounds appear to have independently evolved more than once. For instance, clusters for the biosynthesis of the diterpene defense compound momilactone A have evolved both in cereals and independently in the bryophyte *Calohypnum plumiforme* [24–26]. Other examples include clusters for 5-keto-7,8-epoxy-casbene biosynthesis in *Euphorbiaceae* [27] and the related diterpene 5,10-diketo-casbene, implicated in resistance to bacterial blight in rice [28], and clusters for the biosynthesis of cyanogenic glycoside defense compounds in *Lotus japonicus*, cassava, and sorghum [29]. In other cases, different “flavors” of clusters appear to have arisen and diversified from a common ancestral BGC, as has been shown for cucurbitacin triterpenoids associated with bitterness and defense in the *Cucurbitaceae* (cucumber, melon, and watermelon) [18,30] and for antinutritional and antifungal steroidal glycoalkaloids in the *Solanaceae* (tomato, potato, and eggplant) [31].

The roles of BGC-produced compounds in plant interactions are indicated in Table 1, where known. In some cases (e.g., the noscapine cluster in poppy), the role of the pathway end product(s) in the producing plant, whether in defense or otherwise, is not known. Importantly, numerous nonclustered pathways for defense-related compounds are found in plants, and BGC-produced compounds are known to have other roles in plants, in addition to their protective roles in chemical defense. For instance, benzoxazinoids (defense compounds produced by grasses and some eudicots) have been implicated in regulation of defense responses, flowering time, auxin metabolism, and iron uptake in maize [32]; cyanogenic glycosides serve as nitrogen storage compounds in the rubber tree [33]; and perturbation of the pathway for the oat defense compound avenacin A-1 can result in accumulation of the precursor β-amyrin with associated effects on root epidermal cell patterning [34].

The phenomenon of gene clustering in specialized metabolism is intriguing from an evolutionary perspective, and several hypotheses have been put forward to explain the evolutionary driving forces behind BGC formation in plants. Arguments regarding gene co-inheritance, gene co-expression, and mitigation against accumulation of toxic intermediates have been previously reviewed in relation to plant specialized metabolism in general [6] and discussed specifically with regard to chemical defense pathways [35]. It has been established that plant BGCs have not originated by horizontal gene transfer from microbes but rather by duplication, recruitment, and neofunctionalization of plant genes [6,36]. Clustering of specialized biosynthetic pathways, many of which have evolved relatively recently in evolutionary time, implies that they are under particular selective pressures and are therefore likely to underlie important traits that enhance fitness (e.g., by providing resistance to pests and pathogens). Genomic factors that may contribute to the formation, regulation, and evolution of BGCs include transposable element-mediated recombination [37], chromosomal inversion [38], gene shuffling [39,40], whole genome duplications [41,42], copy number variations of genes within BGCs [43], chromatin modification [44,45], and chromosomal 3D structure [46].

## Clustering facilitates pathway discovery and elucidation

The organization of genes in BGCs in plants has accelerated gene discovery and elucidation of various biosynthetic pathways. In instances where biosynthetic pathway genes are clustered and genome sequences are available, discovery of one gene in a pathway can lead to identification of others, simply by searching for flanking genes with relevant functional annotations. Clustering has thus facilitated delineation of various plant biosynthetic pathways, including complex pathways for alkaloids [31,47] and terpenes [48]. Additionally, once a BGC is discovered in one plant species, similar clusters can in some cases be identified in related species by searching for clustered orthologs or syntenic regions [27,30]. The physical proximity of genes for biosynthetic pathways in plant genomes can also lead to the discovery of unexpected pathway components that would have been difficult to single out based on orthology or gene expression data alone. For example, investigation of the oat avenacin cluster resulted in the identification of a noncanonical sugar transferase required for avenacin biosynthesis that does not belong to the expected UDP-sugar-dependent glycosyltransferase family (UGT1) traditionally associated with plant specialized metabolism [49]. The association of a new gene family with biosynthesis of plant specialized metabolites, whether or not discovered via a gene cluster, can in turn lead to characterization of additional members of that family that may also have functions in plant specialized metabolism [50,51]. Clustering can also facilitate identification of nonenzymatic components associated with metabolic pathways such as transporters and regulators [51,52].

Importantly, gene clustering can facilitate not only elucidation of biosynthetic pathways for known metabolites of interest, but also de novo pathway discovery, complementing other in silico methods based on gene expression and phylogeny. Several examples of the discovery of previously unknown pathways and chemistries based on gene clustering have been reported, including for thalianol and other *Arabidopsis thaliana* root triterpenoids that shape the root microbiome [21], 20-hydroxybetulinic acid, implicated in root and nodule development in the legume *Lotus japonicus* [53], and triterpenoids of unknown function (yossosides) in spinach [50]. Nontargeted genome mining approaches for BGCs have been widely applied in microbes, for example, for antibiotic discovery [54]. Genome mining approaches to detect BGCs are particularly useful for discovery of pathways for compounds that may be produced only in particular plant tissues or under particular conditions, and so may escape detection by metabolite analysis or bioassays. A genome mining approach for BGCs can be employed, for example, for pathway elucidation of defense-related metabolites [55] or bioactive compounds in medicinal plants [56]. Several bioinformatic tools have been developed in recent years for prediction of candidate BGCs in plants [9,13,14]. Where transcriptome data are available, candidate BGCs identified by genome mining can be triaged to identify those that contain co-expressed genes and so are likely to represent active metabolic pathways. For example, co-expression network analysis combined with a genomic survey of neighboring genes has been demonstrated in several studies to be useful for identifying BGCs in *Arabidopsis thaliana* [57]. For defense-related pathways for which expression is induced in response to challenge, genome mining for BGCs can be coupled with analyses of transcriptomic data (e.g., generating co-expression networks) from experiments in which plants are challenged with pathogens, pathogen-associated elicitors, defense-related hormones, or abiotic stresses. While new genes and pathways can be identified and accessed in this way, often with validation of biochemical function in a heterologous host [58,59], understanding the biological roles of newly discovered molecules in the producing plant represents a significant challenge. However, knowledge of the expression profiles of the newly discovered pathway genes and of the fate of the compounds that these pathways produce (for example, secretion from the root) may provide clues as to their possible roles [21]. Where possible, biological function can then be tested by generating plant lines that do not produce the compound(s) of interest by mutation, gene silencing, or gene editing, and evaluating these for altered abiotic/biotic stress tolerance [23,28,60].

## Potential application in crop protection by metabolic engineering of plant BGCs

Elucidation of biosynthetic pathways for defense compounds and other plant metabolites can ultimately lead to practical applications. Several examples of heterologous expression of plant genes comprising biosynthetic pathways have been reviewed previously [59,61] including those in which increased tolerance to pathogens or pests was demonstrated [17,62]. Although the notion of transferring an entire BGC between plant species via genetic engineering is enticing, this is likely to be technically challenging because BGCs typically range from tens to several hundred kilobases in size [6], and the endogenous promoters controlling gene expression would not necessarily drive sufficient or appropriate expression in the heterologous host (although interestingly, the oat avenacin pathway promoters retain their root meristem expression patterns in heterologous plant species, including both monocots and eudicots [34]). A more plausible approach is cloning of individual genes followed by reassembly of the pathway by multigene cloning or sequential gene stacking in the target plant. This will reduce the overall size of the introduced DNA by removal of any irrelevant intervening genes and intergenic regions, while also allowing for optimization of the control of transgene expression using selected promoters and terminators (e.g., to achieve constitutive, induced, or tissue-specific expression). Clearly, such strategies apply to any plant biosynthetic pathway, regardless of whether the genes are clustered or not in the plant of origin.

Improved understanding of how BGCs are regulated may provide insights into new strategies for optimization of coordinate regulation of multistep pathways engineered into other plant species. For example, genome editing for alteration of chromatin structure at a specific BGC locus could allow activation or repression of the entire biosynthetic pathway at one stroke. Two prominent chromatin marks, H2A.Z and H3K27me3, are associated with activation and repression of plant BGCs, respectively [6], thus manipulation of cluster regulation at this level could potentially be achieved by selectively interfering with chromatin remodeling at the cluster locus. Locus-specific epigenetic editing for gene activation/repression with the CRISPR-Cas9 system has already been demonstrated by several studies in mammalian cells via coupling of dCas9 with chromatin-modifying enzymes [63], and BGC activation in filamentous fungi using CRISPR-Cas9 has also recently been reported [64].

Another approach for trait improvement in crops that has been used for decades and does not rely on genetic engineering or genome editing is introgression breeding. Here, wild relatives of crop plants are commonly used as a genetic pool from which beneficial genes are introgressed into the cultivated species, usually with the aim of conferring enhanced pathogen resistance or abiotic stress tolerance [65]. The co-localization of genes in a BGC allows for an entire biosynthetic pathway to be transferred into the cultivated species in a single introgressed segment. In contrast, transfer of a dispersed biosynthetic pathway using such an approach would be difficult. While intentional, breeding-mediated introduction of a clustered biosynthetic pathway has not yet been reported, this is very likely to be possible. Introgression of an acylsugar BGC into tomato from its wild relative *Solanum pennellii*, for example, was shown to increase levels of medium chain acylsugars in trichomes of an isolated tomato introgression line [66].

## Concluding remarks

Since the first report of a BGC in plants more than 20 years ago [67], numerous other examples of such clusters have been identified and characterized. The discovery of these gene clusters has facilitated elucidation of complex metabolic pathways and revealed genetic mechanisms for chemical diversification. It has further enabled the roles of newly discovered BGC pathway products in interactions between plants and other organisms to be shown, as demonstrated by the combined use of gene silencing and plant–pathogen assays [23,28,60]. The inventory of characterized BGCs will inevitably continue to increase as sequencing technologies continue to develop and become cheaper, and more plant genome sequences become available. Key advances include single-molecule long read sequencing, physical mapping technologies such as optical mapping and Hi-C, improved genome assembly algorithms [68], and the establishment of ambitious new initiatives for large-scale sequencing of eukaryote genomes, such as the Earth BioGenome (https://www.earthbiogenome.org/) and Darwin Tree of Life Projects (https://www.darwintreeoflife.org/).

Although much progress has been made with regard to our understanding of BGCs in plants, many questions remain open. One notable question is the extent to which gene clustering occurs in plant metabolism in general, and in chemical defense pathways specifically. Many of the compounds produced by plant BGCs are known to provide protection against pests or pathogens. In other cases, the ecological roles are not known, but the BGC products are important as therapeutic drugs or drug precursors (e.g., noscapine and thebaine). Thus, future discoveries of novel BGCs will provide new insights into the roles of specialized metabolites in interactions between plants and other organisms and may offer solutions for crop improvement through metabolic engineering (e.g., for enhanced abiotic/biotic stress tolerance or optimized production of medicinal compounds). They will also furnish gene sets for the production of drugs and other high value compounds in heterologous expression systems such as yeast and tobacco [58].

## References

[1] Afendi FM, Okada T, Yamazaki M, Hirai-Morita A, Nakamura Y, Nakamura K, et al. KNApSAcK Family Databases: Integrated Metabolite-Plant Species Databases for Multifaceted Plant Research. Plant Cell Physiol. 2012;53(2). doi: 10.1093/pcp/pcr165 WOS:000300497500001. 22123792

[2] Dixon RA, Strack D. Phytochemistry meets genome analysis, and beyond. Phytochemistry. 2003;62(6):815–6. doi: 10.1016/s0031-9422(02)00712-4 WOS:000181400800001. 12590109

[3] Erb M, Kliebenstein DJ. Plant Secondary Metabolites as Defenses, Regulators, and Primary Metabolites: The Blurred Functional Trichotomy. Plant Physiol. 2020;184(1):39–52. Epub 2020/07/07. doi: 10.1104/pp.20.00433 ; PubMed Central PMCID: PMC7479915. 32636341 PMC7479915

[4] Medema MH, Osbourn A. Computational genomic identification and functional reconstitution of plant natural product biosynthetic pathways. Nat Prod Rep. 2016;33(8):951–62. doi: 10.1039/c6np00035e WOS:000381716700006. 27321668 PMC4987707

[5] Nutzmann HW, Osbourn A. Gene clustering in plant specialized metabolism. Curr Opin Biotechnol. 2014;26:91–9. doi: 10.1016/j.copbio.2013.10.009 WOS:000335111500017. 24679264

[6] Nutzmann HW, Huang AC, Osbourn A. Plant metabolic clusters—from genetics to genomics. New Phytol. 2016;211(3):771–89. doi: 10.1111/nph.13981 WOS:000379937200003. 27112429 PMC5449196

[7] Nützmann HW, Scazzocchio C, Osbourn A. Metabolic Gene Clusters in Eukaryotes. Annu Rev Genet. 2018;52:159–83. Epub 2018/09/05. doi: 10.1146/annurev-genet-120417-031237 . 30183405

[8] Boycheva S, Daviet L, Wolfender JL, Fitzpatrick TB. The rise of operon-like gene clusters in plants. Trends Plant Sci. 2014;19(7):447–59. Epub 2014/02/27. doi: 10.1016/j.tplants.2014.01.013 . 24582794

[9] Kautsar SA, Duran HGS, Blin K, Osbourn A, Medema MH. plantiSMASH: automated identification, annotation and expression analysis of plant biosynthetic gene clusters. Nucleic Acids Res. 2017;45(W1):W55–W63. doi: 10.1093/nar/gkx305 WOS:000404427000010. 28453650 PMC5570173

[10] Lee HA, Kim S, Choi D. Expansion of sesquiterpene biosynthetic gene clusters in pepper confers nonhost resistance to the Irish potato famine pathogen. New Phytol. 2017;215(3):1132–43. doi: 10.1111/nph.14637 WOS:000405197500020. 28631815

[11] Boutanaev AM, Moses T, Zi J, Nelson DR, Mugford ST, Peters RJ, et al. Investigation of terpene diversification across multiple sequenced plant genomes. Proc Natl Acad Sci U S A. 2015;112(1):E81–8. Epub 2014/12/10. doi: 10.1073/pnas.1419547112 ; PubMed Central PMCID: PMC4291660. 25502595 PMC4291660

[12] Sarris PF, Cevik V, Dagdas G, Jones JD, Krasileva KV. Comparative analysis of plant immune receptor architectures uncovers host proteins likely targeted by pathogens. BMC Biol. 2016;14:8. Epub 2016/02/19. doi: 10.1186/s12915-016-0228-7 ; PubMed Central PMCID: PMC4759884. 26891798 PMC4759884

[13] Topfer N, Fuchs LM, Aharoni A. The PhytoClust tool for metabolic gene clusters discovery in plant genomes. Nucleic Acids Res. 2017;45(12):7049–63. doi: 10.1093/nar/gkx404 WOS:000404879000015. 28486689 PMC5499548

[14] Schlapfer P, Zhang PF, Wang CA, Kim T, Banf M, Chae L, et al. Genome-Wide Prediction of Metabolic Enzymes, Pathways, and Gene Clusters in Plants. Plant Physiol. 2017;173(4):2041–59. doi: 10.1104/pp.16.01942 WOS:000402054300009. 28228535 PMC5373064

[15] Wisecaver JH, Borowsky AT, Tzin V, Jander G, Kliebenstein DJ, Rokas A. A Global Coexpression Network Approach for Connecting Genes to Specialized Metabolic Pathways in Plants. Plant Cell. 2017;29(5):944–59. Epub 2017/04/13. doi: 10.1105/tpc.17.00009 ; PubMed Central PMCID: PMC5466033. 28408660 PMC5466033

[16] Armah CN, Mackie AR, Roy C, Price K, Osbourn AE, Bowyer P, et al. The membrane-permeabilizing effect of avenacin A-1 involves the reorganization of bilayer cholesterol. Biophys J. 1999;76(1):281–90. doi: 10.1016/S0006-3495(99)77196-1 WOS:000077870700024. 9876141 PMC1302518

[17] Tattersall DB, Bak S, Jones PR, Olsen CE, Nielsen JK, Hansen ML, et al. Resistance to an herbivore through engineered cyanogenic glucoside synthesis. Science. 2001;293(5536):1826–8. doi: 10.1126/science.1062249 WOS:000170894400046. 11474068

[18] Shang Y, Ma YS, Zhou Y, Zhang HM, Duan LX, Chen HM, et al. Biosynthesis, regulation, and domestication of bitterness in cucumber. Science. 2014;346(6213):1084–8. doi: 10.1126/science.1259215 WOS:000345763400035. 25430763

[19] Sohrabi R, Huh JH, Badieyan S, Rakotondraibe LH, Kliebenstein DJ, Sobrado P, et al. In Planta Variation of Volatile Biosynthesis: An Alternative Biosynthetic Route to the Formation of the Pathogen-Induced Volatile Homoterpene DMNT via Triterpene Degradation in Arabidopsis Roots. Plant Cell. 2015;27(3):874–90. doi: 10.1105/tpc.114.132209 WOS:000354640200029. 25724638 PMC4558649

[20] Hen-Avivi S, Savin O, Racovita RC, Lee WS, Adamski NM, Malitsky S, et al. A Metabolic Gene Cluster in the Wheat W1 and the Barley Cer-cqu Loci Determines beta-Diketone Biosynthesis and Glaucousness. Plant Cell. 2016;28(6):1440–60. doi: 10.1105/tpc.16.00197 WOS:000380689400019. 27225753 PMC4944414

[21] Huang ACC, Jiang T, Liu YX, Bai YC, Reed J, Qu BY, et al. A specialized metabolic network selectively modulates Arabidopsis root microbiota. Science. 2019;364(6440):546−+. doi: 10.1126/science.aau6389 WOS:000467631800033. 31073042

[22] Bennett RN, Wallsgrove RM. Secondary metabolites in plant defense-mechanisms. New Phytol. 1994;127(4):617–33. doi: 10.1111/j.1469-8137.1994.tb02968.x WOS:A1994PG82500001. 33874382

[23] Shen S, Peng M, Fang H, Wang Z, Zhou S, Jing X, et al. An Oryza-Specific Hydroxycinnamoyl Tyramine Gene Cluster Contributes to Enhanced Disease Resistance. Sci Bull. 2021. 10.1016/j.scib.2021.03.015 36654123

[24] Shimura K, Okada A, Okada K, Jikumaru Y, Ko KW, Toyomasu T, et al. Identification of a biosynthetic gene cluster in rice for momilactones. J Biol Chem. 2007;282(47):34013–8. doi: 10.1074/jbc.M703344200 WOS:000251145700015. 17872948

[25] Wilderman PR, Xu MM, Jin YH, Coates RM, Peters RJ. Identification of syn-pimara-7,15-diene synthase reveals functional clustering of terpene synthases involved in rice phytoalexin/allelochemical biosynthesis. Plant Physiol. 2004;135(4):2098–105. doi: 10.1104/pp.104.045971 WOS:000223482400023. 15299118 PMC520781

[26] Mao LF, Kawaide H, Higuchi T, Chen MH, Miyamoto K, Hirata Y, et al. Genomic evidence for convergent evolution of gene clusters for momilactone biosynthesis in land plants. Proc Natl Acad Sci U S A. 2020;117(22):12472–80. doi: 10.1073/pnas.1914373117 WOS:000538147800079. 32409606 PMC7275736

[27] King AJ, Brown GD, Gilday AD, Larson TR, Grahama IA. Production of Bioactive Diterpenoids in the Euphorbiaceae Depends on Evolutionarily Conserved Gene Clusters. Plant Cell. 2014;26(8):3286–98. doi: 10.1105/tpc.114.129668 WOS:000345918600007. 25172144 PMC4371829

[28] Zhan C, Lei L, Liu Z, Zhou S, Yang C, Zhu X, et al. Selection of a subspecies-specific diterpene gene cluster implicated in rice disease resistance. Nat Plants. 2020;6(12):1447–54. Epub 2020/12/07. doi: 10.1038/s41477-020-00816-7 . 33299150

[29] Takos AM, Knudsen C, Lai D, Kannangara R, Mikkelsen L, Motawia MS, et al. Genomic clustering of cyanogenic glucoside biosynthetic genes aids their identification in Lotus japonicus and suggests the repeated evolution of this chemical defence pathway. Plant J. 2011;68(2):273–86. doi: 10.1111/j.1365-313X.2011.04685.x WOS:000295836500007. 21707799

[30] Zhou Y, Ma YS, Zeng JG, Duan LX, Xue XF, Wang HS, et al. Convergence and divergence of bitterness biosynthesis and regulation in Cucurbitaceae. Nature Plants. 2016;2(12). doi: 10.1038/nplants.2016.183 WOS:000395793200006. 27892922 PMC5449191

[31] Itkin M, Heinig U, Tzfadia O, Bhide AJ, Shinde B, Cardenas PD, et al. Biosynthesis of Antinutritional Alkaloids in Solanaceous Crops Is Mediated by Clustered Genes. Science. 2013;341(6142):175–9. doi: 10.1126/science.1240230 WOS:000321965300043. 23788733

[32] Zhou S, Richter A, Jander G. Beyond Defense: Multiple Functions of Benzoxazinoids in Maize Metabolism. Plant Cell Physiol. 2018;59(8):1528–37. doi: 10.1093/pcp/pcy064 . 29584935

[33] Selmar D, Lieberei R, Biehl B. Mobilization and utilization of cyanogenic glycosides: the linustatin pathway. Plant Physiol. 1988;86(3):711–6. doi: 10.1104/pp.86.3.711 ; PubMed Central PMCID: PMC1054557. 16665975 PMC1054557

[34] Kemen AC, Honkanen S, Melton RE, Findlay KC, Mugford ST, Hayashi K, et al. Investigation of triterpene synthesis and regulation in oats reveals a role for β-amyrin in determining root epidermal cell patterning. Proc Natl Acad Sci U S A. 2014;111(23):8679–84. Epub 2014/05/27. doi: 10.1073/pnas.1401553111 ; PubMed Central PMCID: PMC4060722. 24912185 PMC4060722

[35] Takos AM, Rook F. Why biosynthetic genes for chemical defense compounds cluster. Trends Plant Sci. 2012;17(7):383–8. doi: 10.1016/j.tplants.2012.04.004 WOS:000306618100001. 22609284

[36] Matsuba Y, Nguyen TTH, Wiegert K, Falara V, Gonzales-Vigil E, Leong B, et al. Evolution of a Complex Locus for Terpene Biosynthesis in Solanum. Plant Cell. 2013;25(6):2022–36. doi: 10.1105/tpc.113.111013 WOS:000322371500013. 23757397 PMC3723610

[37] Boutanaev AM, Osbourn AE. Multigenome analysis implicates miniature inverted-repeat transposable elements (MITEs) in metabolic diversification in eudicots. Proc Natl Acad Sci U S A. 2018;115(28):E6650–E8. doi: 10.1073/pnas.1721318115 WOS:000438050900032. 29941591 PMC6048515

[38] Liu ZH, Cheema J, Vigouroux M, Hill L, Reed J, Paajanen P, et al. Formation and diversification of a paradigm biosynthetic gene cluster in plants. Nat Commun. 2020;11(1). doi: 10.1038/s41467-020-19153-6 WOS:000586505700003. 33097700 PMC7584637

[39] Liu ZH, Duran HGS, Harnvanichvech Y, Stephenson MJ, Schranz ME, Nelson D, et al. Drivers of metabolic diversification: how dynamic genomic neighbourhoods generate new biosynthetic pathways in the Brassicaceae. New Phytol. 2020;227(4):1109–23. doi: 10.1111/nph.16338 WOS:000504608000001. 31769874 PMC7383575

[40] Peters RJ. Doing the gene shuffle to close synteny: dynamic assembly of biosynthetic gene clusters. New Phytol. 2020;227(4):992–4. Epub 2020/05/20. doi: 10.1111/nph.16631 ; PubMed Central PMCID: PMC7856633. 32433781 PMC7856633

[41] Rai A, Hirakawa H, Nakabayashi R, Kikuchi S, Hayashi K, Rai M, et al. Chromosome-level genome assembly of Ophiorrhiza pumila reveals the evolution of camptothecin biosynthesis. Nat Commun. 2021;12(1):405. Epub 2021/01/15. doi: 10.1038/s41467-020-20508-2 . 33452249 PMC7810986

[42] Guo L, Winzer T, Yang XF, Li Y, Ning ZM, He ZS, et al. The opium poppy genome and morphinan production. Science. 2018;362(6412):343–6. doi: 10.1126/science.aat4096 WOS:000447680100049. 30166436

[43] Li QS, Ramasamy S, Singh P, Hagel JM, Dunemann SM, Chen X, et al. Gene clustering and copy number variation in alkaloid metabolic pathways of opium poppy. Nat Commun. 2020;11(1). doi: 10.1038/s41467-020-15040-2 WOS:000544002100001. 32132540 PMC7055283

[44] Nützmann HW, Osbourn A. Regulation of metabolic gene clusters in Arabidopsis thaliana. New Phytol. 2015;205(2):503–10. Epub 2014/11/21. doi: 10.1111/nph.13189 ; PubMed Central PMCID: PMC4301183. 25417931 PMC4301183

[45] Yu N, Nützmann HW, MacDonald JT, Moore B, Field B, Berriri S, et al. Delineation of metabolic gene clusters in plant genomes by chromatin signatures. Nucleic Acids Res 2016;44(5):2255–65. Epub 2016/02/18. doi: 10.1093/nar/gkw100 ; PubMed Central PMCID: PMC4797310. 26895889 PMC4797310

[46] Nutzmann HW, Doerr D, Ramirez-Colmenero A, Sotelo-Fonseca JE, Wegel E, Di Stefano M, et al. Active and repressed biosynthetic gene clusters have distinct chromosome states. Proc Natl Acad Sci U S A. 2020;117(24):13800–9. doi: 10.1073/pnas.1920474117 WOS:000546043800005. 32493747 PMC7306824

[47] Winzer T, Gazda V, He Z, Kaminski F, Kern M, Larson TR, et al. A Papaver somniferum 10-Gene Cluster for Synthesis of the Anticancer Alkaloid Noscapine. Science. 2012;336(6089):1704–8. doi: 10.1126/science.1220757 WOS:000305794500050. 22653730

[48] Li Y, Leveau A, Zhao Q, Feng Q, Lu H, Miao J, et al. Subtelomeric assembly of a multi-gene pathway for antimicrobial defense compounds in cereals. Nat Commun. 12, 2563 (2021). doi: 10.1038/s41467-021-22920-8 33963185 PMC8105312

[49] Orme A, Louveau T, Stephenson MJ, Appelhagen I, Melton R, Cheema J, et al. A noncanonical vacuolar sugar transferase required for biosynthesis of antimicrobial defense compounds in oat. Proc Natl Acad Sci U S A. 2019;116(52):27105–14. doi: 10.1073/pnas.1914652116 WOS:000504656900122. 31806756 PMC6936528

[50] Jozwiak A, Sonawane PD, Panda S, Garagounis C, Papadopoulou KK, Abebie B, et al. Plant terpenoid metabolism co-opts a component of the cell wall biosynthesis machinery. Nat Chem Biol. 2020;16(7):740−+. doi: 10.1038/s41589-020-0541-x WOS:000533828100001. 32424305

[51] Dastmalchi M, Chang L, Chen R, Yu L, Chen X, Hagel JM, et al. Purine Permease-Type Benzylisoquinoline Alkaloid Transporters in Opium Poppy. Plant Physiol. 2019;181(3):916–33. Epub 2019/08/29. doi: 10.1104/pp.19.00565 ; PubMed Central PMCID: PMC6836811. 31467164 PMC6836811

[52] Darbani B, Motawia MS, Olsen CE, Nour-Eldin HH, Møller BL, Rook F. The biosynthetic gene cluster for the cyanogenic glucoside dhurrin in Sorghum bicolor contains its co-expressed vacuolar MATE transporter. Sci Rep. 2016;6:37079. Epub 2016/11/14. doi: 10.1038/srep37079 ; PubMed Central PMCID: PMC5107947. 27841372 PMC5107947

[53] Krokida A, Delis C, Geisler K, Garagounis C, Tsikou D, Pena-Rodriguez LM, et al. A metabolic gene cluster in Lotus japonicus discloses novel enzyme functions and products in triterpene biosynthesis. New Phytol. 2013;200(3):675–90. doi: 10.1111/nph.12414 WOS:000325555400012. 23909862

[54] Zerikly M, Challis GL. Strategies for the Discovery of New Natural Products by Genome Mining. Chembiochem. 2009;10(4):625–33. doi: 10.1002/cbic.200800389 WOS:000264168000003. 19165837

[55] Kliebenstein DJ. Plant defense compounds: systems approaches to metabolic analysis. Annu Rev Phytopathol. 2012;50:155–73. Epub 2012/06/15. doi: 10.1146/annurev-phyto-081211-172950 . 22726120

[56] Kellner F, Kim J, Clavijo BJ, Hamilton JP, Childs KL, Vaillancourt B, et al. Genome-guided investigation of plant natural product biosynthesis. Plant J. 2015;82(4):680–92. doi: 10.1111/tpj.12827 WOS:000354288500012. 25759247

[57] Tohge T, Fernie AR. Co-regulation of Clustered and Neo-functionalized Genes in Plant-Specialized Metabolism. Plants-Basel. 2020;9(5). doi: 10.3390/plants9050622 WOS:000542286900059. 32414181 PMC7285293

[58] Owen C, Patron NJ, Huang A, Osbourn A. Harnessing plant metabolic diversity. Curr Opin Chem Biol. 2017;40:24–30. Epub 2017/05/17. doi: 10.1016/j.cbpa.2017.04.015 ; PubMed Central PMCID: PMC5693780. 28527344 PMC5693780

[59] O’Connor SE. Engineering of Secondary Metabolism. Annu Rev Genet. 2015;49:71–94. doi: 10.1146/annurev-genet-120213-092053 WOS:000367291000004. 26393965

[60] Jeon JE, Kim JG, Fischer CR, Mehta N, Dufour-Schroif C, Wemmer K, et al. A Pathogen-Responsive Gene Cluster for Highly Modified Fatty Acids in Tomato. Cell. 2020;180(1):176−+. doi: 10.1016/j.cell.2019.11.037 WOS:000506574100021. 31923394 PMC6956849

[61] Pyne ME, Narcross L, Martin VJJ. Engineering Plant Secondary Metabolism in Microbial Systems. Plant Physiol. 2019;179(3):844–61. doi: 10.1104/pp.18.01291 WOS:000459688800008. 30643013 PMC6393802

[62] Polturak G, Grossman N, Vela-Corcia D, Dong Y, Nudel A, Pliner M, et al. Engineered gray mold resistance, antioxidant capacity and pigmentation in betalain-producing crops and ornamentals. Proc Natl Acad Sci U S A. 2017;114(34):9062–7. doi: 10.1073/pnas.1707176114 28760998 PMC5576821

[63] Lo A, Qi L. Genetic and epigenetic control of gene expression by CRISPR-Cas systems. F1000Res. 2017;6. Epub 2017/06/27. doi: 10.12688/f1000research.11113.1 ; PubMed Central PMCID: PMC5464239. 28649363 PMC5464239

[64] Roux I, Woodcraft C, Hu JY, Wolters R, Gilchrist CLM, Chooi YH. CRISPR-Mediated Activation of Biosynthetic Gene Clusters for Bioactive Molecule Discovery in Filamentous FungiACS Synth Biol. 2020;9(7):1843–54. doi: 10.1021/acssynbio.0c00197 WOS:000551555500033. 32526136

[65] Zamir D. Improving plant breeding with exotic genetic libraries. Nat Rev Genet. 2001;2(12):983–9. doi: 10.1038/35103590 WOS:000172545800019. 11733751

[66] Fan PX, Wang PP, Lou YR, Leong BJ, Moore BM, Schenck CA, et al. Evolution of a plant gene cluster in Solanaceae and emergence of metabolic diversity. Elife. 2020;9. doi: 10.7554/eLife.56717 WOS:000557388100001. 32613943 PMC7386920

[67] Frey M, Chomet P, Glawischnig E, Stettner C, Grun S, Winklmair A, et al. Analysis of a chemical plant defense mechanism in grasses. Science. 1997;277(5326):696–9. doi: 10.1126/science.277.5326.696 WOS:A1997XN90700044. 9235894

[68] Michael TP, VanBuren R. Building near-complete plant genomes. Curr Opin Plant Biol. 2020;54:26–33. Epub 2020/01/22. doi: 10.1016/j.pbi.2019.12.009 . 31981929

[69] Qi X, Bakht S, Leggett M, Maxwell C, Melton R, Osbourn A. A gene cluster for secondary metabolism in oat: Implications for the evolution of metabolic diversity in plants. Proc Natl Acad Sci U S A. 2004;101(21):8233–8. doi: 10.1073/pnas.0401301101 WOS:000221652000070. 15148404 PMC419586

[70] Field B, Fiston-Lavier AS, Kemen A, Geisler K, Quesneville H, Osbourn AE. Formation of plant metabolic gene clusters within dynamic chromosomal regions. Proc Natl Acad Sci U S A. 2011;108(38):16116–21. doi: 10.1073/pnas.1109273108 WOS:000295030000086. 21876149 PMC3179108

[71] Field B, Osbourn AE. Metabolic diversification—independent assembly of operon-like gene clusters in different plants. Science. 2008;320(5875):543–7. Epub 2008/03/20. doi: 10.1126/science.1154990 . 18356490

[72] Swaminathan S, Morrone D, Wang Q, Fulton DB, Peters RJ. CYP76M7 Is an ent-Cassadiene C11 alpha-Hydroxylase Defining a Second Multifunctional Diterpenoid Biosynthetic Gene Cluster in Rice. Plant Cell. 2009;21(10):3315–25. doi: 10.1105/tpc.108.063677 WOS:000272252100025. 19825834 PMC2782285

[73] Matsuba Y, Zi JC, Jones AD, Peters RJ, Pichersky E. Biosynthesis of the Diterpenoid Lycosantalonol via Nerylneryl Diphosphate in Solanum lycopersicum. PLoS ONE. 2015;10(3). doi: 10.1371/journal.pone.0119302 WOS:000352138500095. 25786135 PMC4364678

[74] Chen X, Hagel JM, Chang LM, Tucker JE, Shiigi SA, Yelpaala Y, et al. A pathogenesis-related 10 protein catalyzes the final step in thebaine biosynthesis. Nat Chem Biol. 2018;14(7):738−+. doi: 10.1038/s41589-018-0059-7 WOS:000435446600020. 29807982

[75] Knoch E, Motawie MS, Olsen CE, Moller BL, Lyngkjaer MF. Biosynthesis of the leucine derived alpha-, beta- and gamma-hydroxynitrile glucosides in barley (Hordeum vulgare L.). Plant J. 2016;88(2):247–56. doi: 10.1111/tpj.13247 WOS:000388442300008. 27337134

